# Full-Stokes Retrieving and Configuration Optimization in a Time-Integration Imaging Polarimeter

**DOI:** 10.3390/s22134733

**Published:** 2022-06-23

**Authors:** Naiting Gu, Bowen Lian, Yawei Xiao, Linhai Huang

**Affiliations:** 1 Institute of Optics and Electronics, Chinese Academy of Sciences, Chengdu 610209, China; bwenlian@163.com (B.L.); hlhjs@163.com (L.H.); 2 The Key Laboratory on Adaptive Optics, Chinese Academy of Sciences, Chengdu 610209, China; 3 University of Chinese Academy of Sciences, Beijing 100049, China

**Keywords:** imaging polarimeter, full-Stokes vector retrieving, time integration, configuration optimization

## Abstract

A time-integration imaging polarimeter with continuous rotating retarder is presented, and its full-Stokes retrieving and configuration optimization are also demonstrated. The mathematical expression between the full-Stokes vector and the time-integration light intensities is derived. As a result, the state of polarization of incident light can be retrieved by only one matrix calculation. However, the modulation matrix deviates from the initial well-conditioned status due to time integration. Thus, we re-optimize the nominal angles for the special retardance of 132° and 90° with an exposure angle of 30°, which results in a reduction of 31.8% and 16.8% of condition numbers comparing to the original configuration, respectively. We also give global optimization results under different exposure angles and retardance of retarder; as a result, the 137.7° of retardance achieves a minimal condition number of 2.0, which indicates a well-conditioned polarimeter configuration. Besides, the frame-by-frame algorithm ensures the dynamic performance of the presented polarimeter. For a general brushless DC motor with a rotating speed of over 2000 rounds per minute, the speed of polarization imaging will achieve up to 270 frames per second. High precision and excellent dynamic performance, together with features of compactness, simplicity, and low cost, may give this traditional imaging polarimeter new life and attractive prospects.

## 1. Introduction

The imaging polarimeter is a kind of important tool because of its special ability of acquiring the state of polarization information of incident light, which contains an object’s special characteristics such as surface features including shapes, roughness and material, etc. [1,2,3]. The imaging polarimeter can provide unique additional valuable information, which may not be captured by traditional intensity-based cameras [4]. Thus, it has been widely used in various scenarios such as target detection [5], material characterization [6,7], remote sensing [8], biomedical imaging [9,10], and astronomy [11,12,13], etc. The existing polarimeter includes four classic types, i.e., division of time polarimeter (DoTP), division of focal plane polarimeter (DoFP), division of amplitude polarimeter (DoAmP) and division of aperture polarimeter (DoAP). The DoTP is the most traditional imaging polarimeter design, which consists of a rotating retarder and a fixed polarizer to measure the full-Stokes vector of incident light [14]. This kind of DoTP collects the total intensity of each polarization component by a photodetector, ensuring the high and uniform signal-to-noise ratio (SNR) and extinction ratio, which make them achieve higher theoretical measuring accuracy [15] and great fit for high-sensitive measuring scenarios [16]. However, it has often been classified as a stationary imaging polarimeter, because the fast axis and its retardance of the used retarders is optimized beforehand, and the light intensities must be detected at the optimal pre-determined angles [17]. It is difficult to operate at high speed due to inaccurate SoP retrieving under continuously high-speed rotating motors with camera readout at the associated high speed. As a result, several minutes will be taken during measurements [18]. Comparing with the DoTP, the DoAmP and the DoAP are both simultaneous imaging polarimeters, which capture multiple images of four sub-beams derived from the light beam under test using four photodetectors or a single camera. Although they promote the real-time property obviously, these kinds of polarimeter are not applied in broad scenes, as limited by their weaknesses such as a complex structure, poor stability, and difficult alignment because of their bulky optical components [19]. The DoFP seems to overcome the above problems, as it utilizes a micro-grid array to obtain the Stokes vector and comes with a compact structure, light weight, and snapshot nature. However, its weaknesses such as fixed-pattern noise (FPN), low and non-uniform extinction ratio, orientation misalignment of micro-polarizer and also being only appliable to linear Stokes components also limit its performance [20,21,22]. Meanwhile, several new kinds of DoTP utilizing photoelastic modulators, liquid-crystal retarders, electro-optical modulators or binary polarization rotators have been proposed [23,24,25,26,27]. However, some disadvantages still hinder their wide application, such as chromatic dispersion, narrow spectral band and temperature sensitivity. Comparatively, the DoTP by rotating polarizer or retarder has good performances in terms of accuracy and sensitivity, except for dynamic property. Thus, some authors have moved back to the research of classic DoTP with a non-stop polarization modulator during polarization imaging, and the Stokes vector is resolved from captured images by utilizing temporal averaging or short integration time methods [28,29,30]. They prompt the development of the DoTP, and these methods bring about considerable improvement on the dynamic performance, while keeping its high accuracy. However, the indirect mathematical presentation, together with a limited integration-time combination of not-best-optimized configuration, hints to the fact that there is still room for improvement.

In this paper, we present a full-Stokes retrieving and configuration optimization method based on a time-integration imaging polarimeter with a continuous rotating retarder and a fixed polarizer. We derived the mathematical expressions between the full-Stokes vector and the time-integration light intensities, and the state of polarization of incident light can be retrieved by only one matrix calculation. To relieve the performance degradation due to time integration, we also re-optimize the nominal angles and retardance of the retarder to minimize the condition number (CN) and realize the best error-propagation performance. The numeric analysis has also been performed, and the results show that the full-Stokes can be retrieved by using the presented method and mathematical model, and the optimization can improve the performance of the presented polarimeter. This kind of polarized optical sensor should have attractive prospects in optical target sensing, biomedical optical imaging, and optical astronomical observation, and so on, due to its high precision, excellent dynamic performance and complete Stokes vector.

## 2. Methods

**Figure 1:**
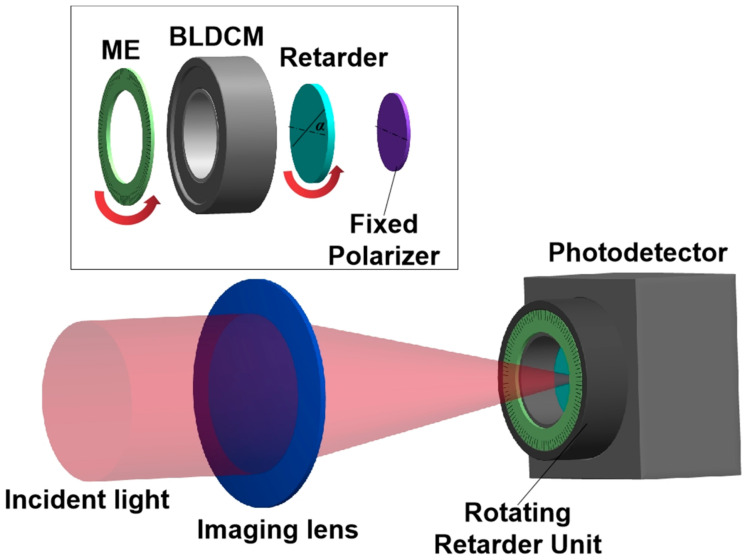
Schematic layout of full-Stokes imaging polarimeter by time integration (α: azimuthal angle of the rotating retarder).

The schematic layout of the time-integration imaging polarimeter is drawn in Figure 1, which consists of an imaging lens, a continuously rotating retarder unit (RRU), a fixed polarizer and a photodetector. The RRU includes a fast-rotating brushless DC motor (BLDCM), an accurate magnetic encoder (ME), a retarder, and a fixed polarizer with a high extinction ratio. The polarizer is jointed tightly on the photosensitive surface of the photodetector, as shown in the illustration of the top side map in Figure 1. The outputting Stokes vector **S***_out_* can be presented when incident polarized light **S***_in_* passes through the rotatable retarder and the fixed polarizer as

(1) $$S_{out}=M_{P}(\theta )M_{R}(\alpha ,\delta )S_{in}$$

where **M***_p_*(*θ*) and **M***_R_*(*α*, *δ*) are the Mueller matrix of the polarizer and retarder, respectively. *θ* presents the angle from horizontal direction to the transmission axis of the polarizer, and (*α*, *δ*) are the azimuthal angle and retardance of the retarder. The value of *θ* will not affect the performance of the imaging polarizer, and it is set as 0° in general for simplification [13]. **S***_in_* = [*I_in_*, *Q_in_*, *U_in_*, *V_in_*] presents the full-Stokes vector of incident light.

Substituting the **M***_p_*(*θ*) and **M***_R_*(*α*, *δ*) into Equation (1), the outputting light intensity after polarization modulation can be expressed as

(2) $$I_{out}=\frac{1}{2}\left[I_{in}+f(\alpha ,\delta )\cdot Q_{in}+g(\alpha ,\delta )\cdot U_{in}+p(\alpha ,\delta )\cdot V_{in}\right]$$

where

(3) $$\begin{array}{l} f(\alpha ,\delta )=\cos^{2}2\alpha +\sin^{2}2\alpha \cos\delta \\ g(\alpha ,\delta )=\sin2\alpha \cos2\alpha (1-\cos\delta )\\ p(\alpha ,\delta )=-\sin2\alpha \sin\delta \end{array}$$

When the retarder rotates continuously, the instantaneous outputting light intensity will also be a time-varying variable. The time-integration light intensity detected by the photodetector during exposure time can be presented by

(4) $$\int_{t_{1}}^{t_{2}}I_{out}(t)dt=\frac{1}{2}\left\{\int_{t_{1}}^{t_{2}}I_{in}dt\right.+Q_{in}\int_{t_{1}}^{t_{2}}f\left[\alpha (t),\delta \right]dt\left.+U_{in}\int_{t_{1}}^{t_{2}}g\left[\alpha (t),\delta \right]dt+V_{in}\int_{t_{1}}^{t_{2}}p\left[\alpha (t),\delta \right]dt\right\}$$

where *t*_1_ and *t*_2_ present the starting and ending times of the photodetector exposure, respectively. *α*(*t*) is the instantaneous azimuthal angle of fast axis of the retarder, which depends on the rotating speed *ω*_0_ of BLDCM and time elapse, and it is

(5) $$\alpha (t)=\omega_{0}t+C$$

where *C* is the constant angle deviation, which has no influence for system optimizing and polarization imaging (*C* = 0 in general).

The time integration of time-varying coefficients of (*Q_in_*, *U_in_*, *V_in_*) are derived in Equations (6)–(8), and they are

(6) ∫t1t2fα(t),δdt=∫t1t2cos22α(t)+sin22α(t)cosδdt=t2−t1−121−cosδ∫t1t2(1−cos4ω0t)dt=12(1+cosδ)(t2−t1)+18ω0(1−cosδ)(sin4ω0t2−sin4ω0t1)

(7) ∫t1t2gα(t),δdt=∫t1t212sin4α(t)(1−cosδ)dt=12(1−cosδ)∫t1t2sin(4ω0t)dt=−18ω0(1−cosδ)(cos4ω0t2−cos4ω0t1)

(8) ∫t1t2pαt,δdt=∫t1t2−sin2α(t)sinδdt=−sinδ∫t1t2sin2ω0tdt=12ω0sinδcos2ω0t2−cos2ω0t1

Define the nominal angle *α*_0_ = *α*(*t*_0_) = *ω*_0_*t*_0_ and the exposure angle ∆*α* = *ω*_0_∆*t*, which present the initial azimuth angle of the retarder and sweeping angle during exposure time ∆*t*, respectively. Let *t*_0_ = *t*_1_ and ∆*t* = *t*_2_ − *t*_1_, then Equations (6)–(8) can be represented by

(9) ∫t1t2fω0t,δdt=18ω04(1+cosδ)Δα+(1−cosδ)sin4(α0+Δα)−sin4α0∫t1t2gω0t,δdt=−18ω0(1−cosδ)cos4(α0+Δα)−cos4α0∫t1t2pω0t,δdt=12ω0sinδcos2(α0+Δα)−cos2α0

For a certain imaging polarimeter, *δ* is a constant parameter for all measurements. The rotating speed *ω*_0_ and the exposure angle ∆*α* can be adjusted according to the requirements of measurement speed and exposure time ∆*t*, which should keep a constant value throughout one time measurement. Substituting Equation (9) into Equation (4), the outputting light intensities for *N* measurements can be represented as

(10) $$\left[\begin{array}{c} I_{out}^{1}\\ I_{out}^{2}\\ \vdots \\ I_{out}^{N} \end{array}\right]=\frac{\Delta \alpha }{2\omega_{0}}\left[\begin{array}{cccc} 1 & F\left(\alpha_{01}\right) & G\left(\alpha_{01}\right) & P\left(\alpha_{01}\right)\\ 1 & F\left(\alpha_{02}\right) & G\left(\alpha_{02}\right) & P\left(\alpha_{02}\right)\\ \vdots & \vdots & \vdots & \vdots \\ 1 & F\left(\alpha_{0N}\right) & G\left(\alpha_{0N}\right) & P\left(\alpha_{0N}\right) \end{array}\right]\left[\begin{array}{c} I_{in}\\ Q_{in}\\ U_{in}\\ V_{in} \end{array}\right]$$

where *I_out_* presents the time integration of *I_out_*(*t*) during the photodetector exposure, and *α*_0*i*_ is the *i*th nominal angle. *F*(*α*_0*i*_), *G*(*α*_0*i*_) and *P*(*α*_0*i*_) are expressed as

(11) $$\begin{array}{l} F\left(\alpha_{0i}\right)=\frac{1+\cos\delta }{2}+\frac{\left(1-\cos\delta \right)}{8}\frac{\sin4\left(\alpha_{0i}+\Delta \alpha \right)-\sin4\alpha_{0i}}{\Delta \alpha }\\ G\left(\alpha_{0i}\right)=-\frac{1-\cos\delta }{8}\frac{\cos4\left(\alpha_{0i}+\Delta \alpha \right)-\cos4\alpha_{0i}}{\Delta \alpha }\\ P\left(\alpha_{0i}\right)=\frac{\sin\delta }{2}\frac{\cos2\left(\alpha_{0i}+\Delta \alpha \right)-\cos2\alpha_{0i}}{\Delta \alpha } \end{array}$$

In fact, Equation (10) builds a new relationship between the full-Stokes vector of incident light and the corresponding detected light intensities with time integration. In the equation, the modulation matrix is decided by the retardance (*δ*) and nominal angle serial (*α*_0*i*_) of the used retarder, the rotating speed of BLDCM (*ω*_0_) and the exposure time of the photodetector (∆*t*), as shown in Equation (11). The nominal angles decide the trigger points of photodetector exposure, and their values will affect the performance of the presented imaging polarimeter. In particular, Equation (11) can be treated as a serial of differential equations when the exposure angle is small enough, and then it will go back to static polarization modulation, as shown in Equation (3).

As stated above, the exposure angle is identical for each nominal angle, and thus it should not be greater than the smallest angle gap between the optimal nominal angles, i.e.,

(12) $$\Delta \alpha \leq \overset{N}{\underset{i=1}{\min}}\left\{\alpha_{0,i+1}-\alpha_{0,i}\right\}$$

In practical applications, the photodetector exposure ∆*t* depends on the intensity of incident light and the speed of system transmission. Thus, Equation (12) hints that the maximum rotating speed of the retarder should satisfy.

(13) $$\omega_{0}\leq \frac{\overset{N}{\underset{i=1}{\min}}\left\{\alpha_{0,i+1}-\alpha_{0,i}\right\}}{\Delta t}$$

## 3. Results

For acquiring better performance, the polarization modulation matrix must be optimized as far from singular as possible, i.e., well conditioned. For a static full-Stokes imaging polarimeter, Sabatke et al. [31] suggested to set up a retarder with retardance of 132° and four azimuthal angles of (±15.1°, ±51.7°), which leads to a condition number (CN) of the polarization modulation matrix close to its theoretical minimization value of 1.7321. To avoid the customization of the retarder, Ambirajan et al. [32] analyzed the optimized azimuthal angles of (−90°, −45°, 30°, 60°) based on a standard quarter-wave plate, which results in a greater CN of 3.6268. In fact, there exist other better azimuthal angles of (14.95°, 50.56°, 129.58°, 165.25°) with a smaller CN of 3.2739 for a standard quarter-wave plate based on our analysis result. Thus, the retardance of 132° and 90° are chosen to analyze the performance of the proposed polarimeter. However, these optimal retardance and azimuthal angles are all based on static configuration.

**Figure 2:**
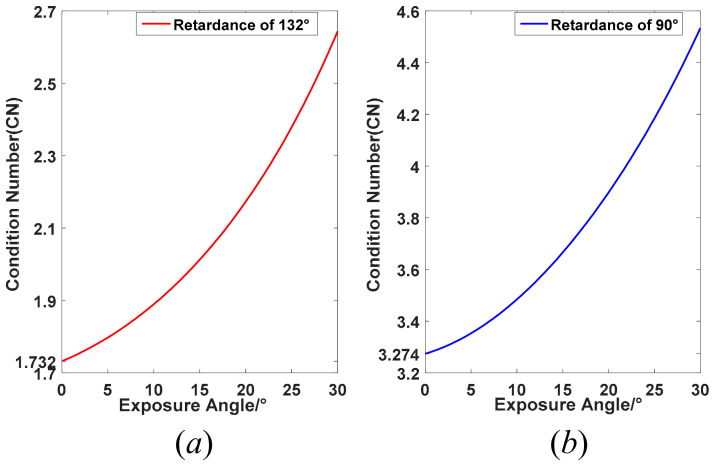
CN deviation from its well-conditioned value with increasing of the exposure angles. (**a**) retardance of 132°; (**b**) retardance of 90°.

**Figure 3:**
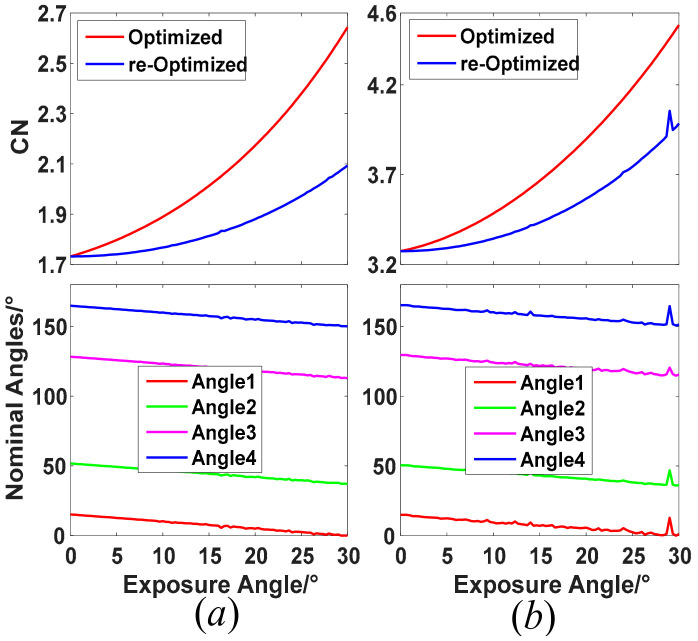
Re-optimized CN and its corresponding nominal angles. (**a**) retardance of 132°; (**b**) retardance of 90°.

For the presented time-integration imaging polarimeter, the CN may deviate from its best-optimized value when the retarder is continuously rotating. According to the above descriptions, we chose two kinds of retarders to analyze the CN performance change with changing of exposure angles, including the best-optimized and customized retardance of 132° and the retardance of 90° generated from a standard quarter-wave plate. Figure 2 shows the changing of CN from its best-optimization configuration when the retardances of the retarder are 132° and 90°, respectively. Here, the largest exposure angle is up to 30°, which depends on the smallest angle gap between the optimal nominal angles, as illustrated in Equation (12). As shown in Figure 2, the CN of Ambirajan’s configuration is increasing from its well-conditioned value with increasing of the exposure angle. The maximum CN is about 2.644 with about a 52.7% increase when the exposure angle is up to 30°. Similarly, the CN will also deviate from the best-optimized value when a standard quarter-wave plate is used as the retarder, and the maximum CN is about 4.534 with a 38.5% increase. This indicates that the performance of these kinds of time-integration imaging polarimeter will become worse, and the deviation relative to their optimized configuration is larger under a greater exposure angle, regardless of its retardance. In fact, the exposure angle can be classified as one of the system parameters of imaging and re-optimize the nominal angles of the retarder for each different exposure angle. Figure 3 gives the re-optimized CN and their corresponding nominal angles for two retardance of 132° and 90°, respectively. As seen from Figure 3, the CN after re-optimization is also increasing with increasing of the exposure angle, but the increasing speed is obviously slower than the initial optimized configuration. The maximum CNs of the retardance of 132° and 90° are 2.094 and 3.985, with 20.9% and 21.7% increases, respectively, which are significantly smaller than before. Apparently, the nominal angles after re-optimization are also adjusted, displaying an almost linear trend. One can build a lookup table (LUT) under these two different retarders, respectively, and it is convenient to find the four best-optimized nominal angles for different exposure angles when the rotating speed of the retarder or the exposure time of the photodetector is settled. It can be concluded easily that the performance of the imaging polarimeter with retardance of 132° is better than 90°, regardless of what the exposure angle is, with about 100% performance improvement for almost all exposure angles. However, the imaging polarimeter with 90° retardance is more achievable and has a lower cost, since most standard retarders are designed at quarter- or half-wave retardance. Besides, the CN is larger obviously than neighbor points when the exposure angle is about 28°, as shown in Figure 3b, which can be treated as a singular point of this kind of polarimeter configuration.

**Figure 4:**
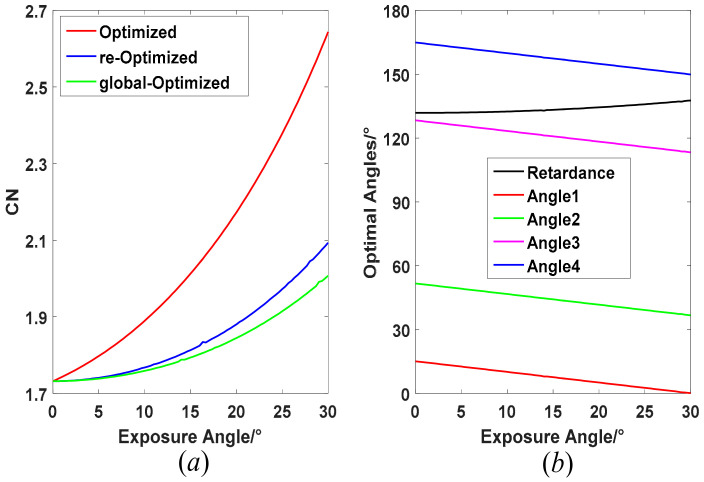
Global-optimized CN and its corresponding retardance and four nominal angles. (**a**) global-optimized CN; (**b**) global-optimized retardance and four nominal angles.

Certainly, one can customize a retarder with special retardance for better performance of an imaging polarimeter, and the 132° may not be the best retardance when the exposure angle exists. In the following optimization, we release the retardance *δ* as an optimal variable, and Equation (10) can be optimized further for a particular exposure angle, i.e., global optimization. Figure 4 gives the comparison of CN curves of initial optimization, re-optimization, and global optimization. The CN of the global-optimized imaging polarimeter is also increased with increasing of the exposure angle, which is similar to others, but its value remains the smallest among these three kinds of configurations for all exposure angles. The maximum CN of global optimization is up to 2.007 with about a 15.9% increase, which is 5% lower than that of the re-optimization configuration. The corresponding best retardance is also increasing slowly, starting from initial retardance of 131.8° to 137.7° with an increasing exposure angle, as shown in Figure 4b. Once the exposure angle is settled, a retarder with global-optimized retardance can be customized for the selected spectral wavelength, and the corresponding four nominal angles are determined as a result.

Based on above analysis, it is suggested to implement a constant exposure angle of an imaging polarimeter to avoid adjusting the nominal angles and even retardance of the retarder frequently. The exposure angle is determined by the rotating speed *ω*_0_ of the retarder and the exposure time ∆*t* of the used photodetector. In a practical imaging polarimeter, the ∆*t* mainly depends on the target brightness and photodetector performance. Thus, the rotating speed of the BLDCM must be adjusted to keep the same exposure angle for different exposure times. As seen from Figure 3b and Figure 4a, a smaller exposure angle performs better, regardless of which kind of configuration is used. However, for a certain exposure time, a smaller exposure angle means a slower rotating speed of the retarder, which will limit the measurement speed of the imaging polarimeter. We suggest that a maximum exposure angle of 30° should be used in a time-integration imaging polarimeter to obtain the best dynamic performance, while scarifying performance slightly of error propagation. The corresponding well-conditioned retardance and nominal angles are listed in Table 1 for customized and quarter-wave plates.

It is not very hard to buy or customize a BLDCM with a high rotating speed of over 2000 rounds per minute (RPM) with ±5 rpm (in our experimental test, the fastest speed of the customized BLDCM was over 2500 rpm), and that means that the shortest time consumed of one measurement of a full-Stokes polarization image is down to 15 ms. If neighboring frames are used, the practical maximum measurement speed of polarization imaging is up to 270 frames per second, under which condition the time-integration imaging polarimeter is capable for dynamic scenes. Besides, the digital bit of commercial ME is over 20 bits, and the angle resolution is greater than 1.2 arc second, which is enough to control the nominal angles and trigger the photodetector. Certainly, for minimizing vibration, the rotatable component should be adjusted carefully to keep a good performance of dynamic balance, and it should also be fixed together with the camera on a rigid structure in a practical application.

## 4. Discussions and Conclusions

In this paper, we have presented a time-integration imaging polarimeter with a continuous rotating retarder. The full-Stokes vector can be retrieved by only one matrix calculation. We also re-optimized the best nominal angles of the imaging polarimeter considering different exposure angles, and the condition numbers for 132° and 90° retardance are maximum reduced, 31.8% and 16.8%, respectively. The performance can be improved further to 36.8% given a customized retarder of 137.7° retardance at maximum exposure angle, which is different obviously with the existing reference. It may cost a lot both in terms of time and financially when one customizes a retarder with a retardance of 137.7°. In fact, there is another way to achieve the special retardance including 137.7° by combining two standard quarter-wave plates with a different fast axis [17].

Overall, the application of time integration releases the dynamic performance of the traditional imaging polarimeter. The maximum measurement speed of the presented imaging polarimeter can achieve up to 270 frames per second when a fast brushless DC motor with a rotating speed of over 2000 rounds per minute is employed to rotate the retarder. Excellent performance comes with advantages, including good dynamic property, compactness, simplicity, high accuracy and low cost, which may give this kind of traditional imaging polarimeter new life and attractive prospects. Certainly, we or other people who are interested in this work should conduct experimental validation in future work, including experimental validations of mathematical models, the best-optimized configuration, and so on.

## Figures and Tables

**Table 1:** Optimized retardance and nominal angles of the used retarder under the suggested maximum exposure angle of 30° in a time-integration imaging polarimeter (unit: °).

Variables	Re-Optimization	Global Optimization
*δ*	90.0	137.7
*α*_01_	1.13	0.12
*α*_02_	36.23	36.70
*α*_03_	115.50	113.31
*α*_04_	151.32	149.88
*CN*	3.985	2.007

## Data Availability

The data that support the findings of this study are available from the first corresponding author upon reasonable request.
